# Supplementary material for: Modeling glioblastoma heterogeneity as a dynamic network of cell states
Source: Mol Syst Biol. 2021 Sep 16;17(9):e10105. doi: 10.15252/msb.202010105 (PMC8444284; doi:10.15252/msb.202010105)
Supplement: Supplementary file 6 — Source Data for Figure 5 [file MSB-17-e10105-s004.zip › Figure5A_sourcedata/GSEA_3017/nosigdb_stateA.GseaPreranked.1622795026485/RICHARDS_NOWAKOWSKI_SCIENCE_2017_RG_EARLY_UPREG.html]

Details for gene set RICHARDS\_NOWAKOWSKI\_SCIENCE\_2017\_RG\_EARLY\_UPREG[GSEA]

|  || Dataset | state53017 |
| Phenotype | NoPhenotypeAvailable |
| Upregulated in class | na\_pos |
| GeneSet | RICHARDS\_NOWAKOWSKI\_SCIENCE\_2017\_RG\_EARLY\_UPREG |
| Enrichment Score (ES) | 0.35907206 |
| Normalized Enrichment Score (NES) | 2.7726445 |
| Nominal p-value | 0.0 |
| FDR q-value | 0.0 |
| FWER p-Value | 0.0 |
Table: GSEA Results Summary

  

Fig 1: Enrichment plot: RICHARDS\_NOWAKOWSKI\_SCIENCE\_2017\_RG\_EARLY\_UPREG      
 Profile of the Running ES Score & Positions of GeneSet Members on the Rank Ordered List

  

| PROBE | GENE SYMBOL | GENE\_TITLE | RANK IN GENE LIST | RANK METRIC SCORE | RUNNING ES | CORE ENRICHMENT || 1 | CDK1 |  |  | 3 | 1.006 | 0.0169 | Yes |
| 2 | MKI67 |  |  | 4 | 0.926 | 0.0357 | Yes |
| 3 | KIF11 |  |  | 6 | 0.870 | 0.0522 | Yes |
| 4 | UBE2C |  |  | 7 | 0.793 | 0.0682 | Yes |
| 5 | KIF20B |  |  | 10 | 0.749 | 0.0811 | Yes |
| 6 | KIF5B |  |  | 16 | 0.687 | 0.0892 | Yes |
| 7 | PPA1 |  |  | 19 | 0.668 | 0.1005 | Yes |
| 8 | HIST1H4C |  |  | 21 | 0.659 | 0.1127 | Yes |
| 9 | RPS24 |  |  | 23 | 0.649 | 0.1247 | Yes |
| 10 | ASPM |  |  | 27 | 0.639 | 0.1341 | Yes |
| 11 | PGAM1 |  |  | 35 | 0.608 | 0.1384 | Yes |
| 12 | CCNB1 |  |  | 37 | 0.599 | 0.1493 | Yes |
| 13 | CKS2 |  |  | 40 | 0.587 | 0.1589 | Yes |
| 14 | NCAPG |  |  | 44 | 0.556 | 0.1667 | Yes |
| 15 | VIM |  |  | 46 | 0.543 | 0.1766 | Yes |
| 16 | CKS1B |  |  | 50 | 0.530 | 0.1839 | Yes |
| 17 | DBF4 |  |  | 55 | 0.514 | 0.1897 | Yes |
| 18 | RPL22L1 |  |  | 56 | 0.512 | 0.2001 | Yes |
| 19 | KIF14 |  |  | 59 | 0.503 | 0.2079 | Yes |
| 20 | CENPE |  |  | 62 | 0.496 | 0.2157 | Yes |
| 21 | MIS18BP1 |  |  | 64 | 0.495 | 0.2246 | Yes |
| 22 | CCNA2 |  |  | 65 | 0.495 | 0.2346 | Yes |
| 23 | TPX2 |  |  | 66 | 0.488 | 0.2445 | Yes |
| 24 | DLGAP5 |  |  | 76 | 0.475 | 0.2437 | Yes |
| 25 | HIST1H1B |  |  | 77 | 0.475 | 0.2533 | Yes |
| 26 | UBE2T |  |  | 83 | 0.470 | 0.2571 | Yes |
| 27 | SAR1A |  |  | 85 | 0.468 | 0.2654 | Yes |
| 28 | DUT |  |  | 87 | 0.466 | 0.2737 | Yes |
| 29 | CENPF |  |  | 94 | 0.461 | 0.2761 | Yes |
| 30 | TOP2A |  |  | 95 | 0.457 | 0.2854 | Yes |
| 31 | HIST1H1D |  |  | 98 | 0.456 | 0.2923 | Yes |
| 32 | ZWINT |  |  | 106 | 0.447 | 0.2933 | Yes |
| 33 | TGIF1 |  |  | 107 | 0.447 | 0.3023 | Yes |
| 34 | ZEB1 |  |  | 109 | 0.446 | 0.3102 | Yes |
| 35 | KIF23 |  |  | 116 | 0.436 | 0.3121 | Yes |
| 36 | BUB1B |  |  | 129 | 0.421 | 0.3068 | Yes |
| 37 | HELLS |  |  | 135 | 0.417 | 0.3095 | Yes |
| 38 | RTKN2 |  |  | 141 | 0.413 | 0.3121 | Yes |
| 39 | CCNB2 |  |  | 147 | 0.411 | 0.3146 | Yes |
| 40 | DNAJC1 |  |  | 151 | 0.407 | 0.3194 | Yes |
| 41 | ANLN |  |  | 152 | 0.407 | 0.3277 | Yes |
| 42 | MAD2L1 |  |  | 153 | 0.406 | 0.3359 | Yes |
| 43 | PBX3 |  |  | 157 | 0.404 | 0.3406 | Yes |
| 44 | ARHGAP11A |  |  | 162 | 0.399 | 0.3441 | Yes |
| 45 | RPS23 |  |  | 163 | 0.399 | 0.3522 | Yes |
| 46 | KIF2C |  |  | 165 | 0.397 | 0.3591 | Yes |
| 47 | NDC80 |  |  | 173 | 0.393 | 0.3590 | No |
| 48 | DCLRE1C |  |  | 190 | 0.381 | 0.3482 | No |
| 49 | KIF22 |  |  | 197 | 0.377 | 0.3489 | No |
| 50 | CDCA3 |  |  | 203 | 0.372 | 0.3507 | No |
| 51 | NFIL3 |  |  | 204 | 0.371 | 0.3582 | No |
| 52 | SMC4 |  |  | 217 | 0.367 | 0.3518 | No |
| 53 | SPC25 |  |  | 226 | 0.363 | 0.3500 | No |
| 54 | H2AFZ |  |  | 245 | 0.351 | 0.3363 | No |
| 55 | TTK |  |  | 262 | 0.340 | 0.3248 | No |
| 56 | KNSTRN |  |  | 263 | 0.340 | 0.3317 | No |
| 57 | SNRPE |  |  | 271 | 0.338 | 0.3304 | No |
| 58 | RPL18A |  |  | 279 | 0.334 | 0.3291 | No |
| 59 | CACYBP |  |  | 288 | 0.330 | 0.3266 | No |
| 60 | HSPD1 |  |  | 291 | 0.328 | 0.3309 | No |
| 61 | NPM1 |  |  | 292 | 0.328 | 0.3375 | No |
| 62 | MND1 |  |  | 304 | 0.324 | 0.3314 | No |
| 63 | RPS6 |  |  | 306 | 0.324 | 0.3368 | No |
| 64 | CENPN |  |  | 310 | 0.323 | 0.3399 | No |
| 65 | HSPB1 |  |  | 312 | 0.322 | 0.3453 | No |
| 66 | PEG3 |  |  | 335 | 0.314 | 0.3263 | No |
| 67 | CREB3L2 |  |  | 348 | 0.310 | 0.3187 | No |
| 68 | PRC1 |  |  | 350 | 0.310 | 0.3238 | No |
| 69 | RPS3 |  |  | 356 | 0.307 | 0.3243 | No |
| 70 | HSP90AB1 |  |  | 363 | 0.306 | 0.3236 | No |
| 71 | ECT2 |  |  | 365 | 0.305 | 0.3286 | No |
| 72 | BRCA1 |  |  | 373 | 0.302 | 0.3266 | No |
| 73 | NET1 |  |  | 378 | 0.301 | 0.3281 | No |
| 74 | CKAP2 |  |  | 379 | 0.301 | 0.3342 | No |
| 75 | RRM2 |  |  | 383 | 0.300 | 0.3368 | No |
| 76 | CENPW |  |  | 386 | 0.299 | 0.3406 | No |
| 77 | BNIP3 |  |  | 395 | 0.296 | 0.3373 | No |
| 78 | HMGA1 |  |  | 397 | 0.295 | 0.3421 | No |
| 79 | RACGAP1 |  |  | 399 | 0.293 | 0.3469 | No |
| 80 | HMGB1 |  |  | 403 | 0.292 | 0.3494 | No |
| 81 | CREB5 |  |  | 407 | 0.291 | 0.3518 | No |
| 82 | HMGB2 |  |  | 424 | 0.288 | 0.3392 | No |
| 83 | NUSAP1 |  |  | 430 | 0.286 | 0.3392 | No |
| 84 | RPL41 |  |  | 436 | 0.285 | 0.3392 | No |
| 85 | CDC25C |  |  | 443 | 0.282 | 0.3380 | No |
| 86 | ANP32E |  |  | 453 | 0.280 | 0.3333 | No |
| 87 | ODC1 |  |  | 460 | 0.279 | 0.3320 | No |
| 88 | HSPE1 |  |  | 465 | 0.277 | 0.3330 | No |
| 89 | ATAD2 |  |  | 470 | 0.275 | 0.3340 | No |
| 90 | EZH2 |  |  | 471 | 0.275 | 0.3396 | No |
| 91 | RPS12 |  |  | 479 | 0.274 | 0.3370 | No |
| 92 | KIF4A |  |  | 504 | 0.269 | 0.3148 | No |
| 93 | ANAPC16 |  |  | 515 | 0.268 | 0.3087 | No |
| 94 | HAUS8 |  |  | 518 | 0.267 | 0.3118 | No |
| 95 | RPL37 |  |  | 525 | 0.266 | 0.3103 | No |
| 96 | ZFP36L1 |  |  | 532 | 0.264 | 0.3087 | No |
| 97 | KIF15 |  |  | 538 | 0.263 | 0.3083 | No |
| 98 | CDKN3 |  |  | 539 | 0.263 | 0.3136 | No |
| 99 | VEGFA |  |  | 546 | 0.261 | 0.3119 | No |
| 100 | RPL23 |  |  | 552 | 0.259 | 0.3114 | No |
| 101 | DDX39A |  |  | 562 | 0.258 | 0.3063 | No |
| 102 | SOX9 |  |  | 577 | 0.254 | 0.2953 | No |
| 103 | PEA15 |  |  | 668 | -0.281 | 0.1972 | No |
| 104 | CKB |  |  | 676 | -0.287 | 0.1949 | No |
| 105 | GPC4 |  |  | 687 | -0.295 | 0.1893 | No |
| 106 | RPS27L |  |  | 688 | -0.295 | 0.1953 | No |
| 107 | ZFHX4 |  |  | 700 | -0.301 | 0.1887 | No |
| 108 | C1orf61 |  |  | 733 | -0.328 | 0.1585 | No |
| 109 | HES1 |  |  | 736 | -0.331 | 0.1629 | No |
| 110 | PON2 |  |  | 743 | -0.338 | 0.1628 | No |
| 111 | HEY1 |  |  | 751 | -0.344 | 0.1617 | No |
| 112 | BOC |  |  | 766 | -0.359 | 0.1528 | No |
| 113 | ITGB8 |  |  | 774 | -0.363 | 0.1521 | No |
| 114 | TPM1 |  |  | 784 | -0.369 | 0.1492 | No |
| 115 | PNRC1 |  |  | 786 | -0.371 | 0.1556 | No |
| 116 | ATF3 |  |  | 836 | -0.428 | 0.1077 | No |
| 117 | ZFP36L2 |  |  | 848 | -0.443 | 0.1040 | No |
| 118 | MAP1B |  |  | 851 | -0.444 | 0.1107 | No |
| 119 | FTH1 |  |  | 910 | -0.573 | 0.0554 | No |
| 120 | TTYH1 |  |  | 932 | -0.652 | 0.0444 | No |
| 121 | CNN3 |  |  | 961 | -0.883 | 0.0300 | No |
Table: GSEA details [plain text format]

  

Fig 2: RICHARDS\_NOWAKOWSKI\_SCIENCE\_2017\_RG\_EARLY\_UPREG: Random ES distribution      
 Gene set null distribution of ES for **RICHARDS\_NOWAKOWSKI\_SCIENCE\_2017\_RG\_EARLY\_UPREG**
